# Relational needs satisfaction scale: adaptation to the Bosnian language and psychometric testing

**DOI:** 10.3389/fpsyg.2025.1528926

**Published:** 2025-04-16

**Authors:** Lejla Mustoo-Başer, Emina Sinanović, Selvira Draganović, Gregor Žvelc

**Affiliations:** ^1^Department of Psychology, Faculty of Arts and Social Sciences, International University of Sarajevo, Sarajevo, Bosnia and Herzegovina; ^2^Department of Psychology, Faculty of Philosophy, University of Sarajevo, Sarajevo, Bosnia and Herzegovina; ^3^Department of Psychology, Faculty of Arts, University of Ljubljana, Ljubljana, Slovenia

**Keywords:** relational needs satisfaction scale, relational needs, relationship satisfaction, instrument adaptation, psychometric properties

## Abstract

**Background:**

Relating to others and establishing relationships is necessary for optimal human functioning. Perceived relational satisfaction appears to be one of the most important aspects of individuals’ lives, reflecting the extent to which our relational needs are met. This study aimed to test the factor structure, item characteristics, and convergent validity of the Bosnian adaptation of the Relational Needs Satisfaction Scale (RNSS).

**Method:**

A total of 420 participants (*N* = 420) completed the Relational Needs Satisfaction Scale (RNSS), the Relationships Questionnaire (RQ), and the Need for Drama Questionnaire (NFD). Descriptive statistics, reliability, and item analysis for the RNSS were conducted. Both exploratory and confirmatory factor analyses were conducted, along with a comparison of four models. The convergent validity of the RNSS was assessed by examining its association with two reference measures and their subscales: RQ and NFD.

**Results:**

The study showed that the translation was adequate, and the Bosnian version of the RNSS proved to be a reliable measure with mostly adequate item parameters. It confirmed that the RNSS structure can be interpreted as a five-factor model, comprising five dimensions and one higher-order factor, as well as a bi-factor model, where the variance of the items is simultaneously explained by a general factor and the five dimensions to varying degrees. The comparison of models and theory indicated the superiority of the bi-factor model.

**Conclusion:**

The adaptation of the RNSS Bosnian version demonstrated content validity, adequate measurement accuracy, and appropriate construct validity, as supported by confirmatory factor analysis. Furthermore, this study provides evidence that the translated RNSS is a valid and reliable instrument.

## 1 Introduction

As one of the most important human motivations, individuals exhibit an innate desire for interpersonal relationships, and the importance of belonging is a universal human need (Allen et al., 2022). Relationships can either enhance or diminish a person’s sense of self and well-being (Scott et al., 2022; White and Jha, 2023). The clarity of self-concept mediates the connection between the satisfaction of psychological needs and values, which lie at the core of individual identity (Russo et al., 2021), with relational identity contributing to life satisfaction (Kreuzbauer et al., 2014).

Relational well-being appears to be predicted by the motivation to sustain relationships and the specific activities that differ based on attachment and personality dimensions (Gaine and La Guardia, 2009). However, fulfilling relationship needs predicts both relational and individual well-being, while relational exclusion significantly contributes to ill-being (Valcke et al., 2020). For example, experiences of relational disequilibrium predicted lower well-being 6 months later (Kumashiro et al., 2008). Additionally, relationship outcomes may be uniquely predicted by perceived relatedness (Patrick et al., 2007).

Furthermore, relatedness, together with autonomy, competence, and authenticity, is associated with daily self-esteem reports while controlling for contributions from unpleasant and pleasant affect (Heppner et al., 2008). However, loneliness appears to mediate the relationship between well-being, particularly life satisfaction, and the unmet need to belong (Mellor et al., 2008).

Although culture and its cross-cultural differences play a crucial role in relational motivation (Yi et al., 2014), the effects of need frustration and satisfaction appear to be consistent across cultures, with no identified moderating role of individual differences (Chen et al., 2015).

In addition, relational entitlement sense (a term related to relational satisfaction) is associated with outcomes in romantic relationships (Candel and Turliuc, 2019), where relational frustration of need-relatedness, competence, and autonomy appears to be linked with demanding tendencies and contributes to feelings of anger, sadness, and fear (Vanhee, 2017). Moreover, individuals with unfulfilled relational needs in romantic relationships, who also have high attachment avoidance, report greater amounts of sexual nostalgia (Muise et al., 2020). Furthermore, according to the study by Tolmacz et al. (2022), lower relational needs satisfaction appears to be associated with disordered eating patterns.

Satisfaction of relational needs relies on key principles of attachment theory, where the template for relationships and general interactions with others is formed through initial emotional connections with primary caregivers and significant others (Fletcher and Gallichan, 2016). Relational support is crucial for both exploratory and attachment needs (Feeney and Collins, 2019). The need for idealization, twinship, and mirroring is highlighted as the main self-object needs that develop as individuals strive to maintain a cohesive self, according to the self-psychology framework (Kohut, 1977). Moreover, the need for relationships is emphasized as a central human motivation in both object relations theory and transactional analysis (Berne, 2016; Winnicot, 1986/1960).

The Relational Needs Model was developed by Erskine (1998, 2015) using the relationally-focused integrative psychotherapy and transactional analysis. This model highlights the importance of relational needs in interpersonal contact and the psychotherapy process, emphasizing their primary influence on an individual’s well-being throughout their lifespan. Relational needs do not refer to physiological needs (e.g., air, appropriate temperature, food); instead, they are specific to interpersonal contact, where satisfaction contributes to the formation of a positive sense of self-in-relationship (Erskine, 2011). If relational needs remain unsatisfied for prolonged periods, feelings of anger, aggression, anxiety, emptiness, frustration, loneliness, nervousness, a sense of insecurity, and overall emotional disturbance may arise (Erskine, 1998; Erskine et al., 1999). We may develop script beliefs such as “I am not important” as a cognitive defense against a lack of relational contact (Erskine, 1980). Consequently, we form implicit relational patterns to compensate for disruptions in relationships, such as insecure attachment styles. Conversely, meeting relational needs can evoke and enhance capacities for intimacy, expansiveness, and creativity (Erskine, 2015).

The eight relational needs are described by Erskine et al. (1999), Erskine (2011), and Erskine (2015).

1. *The need for security* involves recognizing that our feelings are natural and human. It is a visceral experience that includes protecting our emotional and physical vulnerabilities while fostering harmony with others.
2. *The need to feel validated, affirmed, and significant in a relationship* involves recognizing the role and importance of one’s intrapsychic processes, along with the essential part of emotions in both interpersonal and intrapsychic communication.
3. *The need for acceptance by a stable, dependable, and protective person* represents an essential relational need. It signifies the presence of significant others from whom we receive protection, information, guidance, and encouragement. This suggests that the need for intrapsychic protection may be expressed through idealization and, therefore, may not necessarily be considered pathological.
4. *The need for confirmation of personal experience* is also an essential relational need for mutuality. It involves having someone who phenomenologically understands the situation due to similar experiences and a desire to be in the company of someone similar.
5. *The need for self-definition* refers to communicating one’s chosen identity through the expression of interests, ideas, and preferences without facing rejection or humiliation. It involves being different from others and unique while being acknowledged and respected for this uniqueness.
6. *The need to have an impact on another person* refers to an individual’s sense of agency, attracting the other person’s interest and attention and affecting a change in behavior or affect in them.
7. *The need for another person to initiate* refers to having someone else make contact and engage in an exchange. In healthy reciprocal relationships, both individuals act as initiators, acknowledging and validating each other’s investment in the relationship.
8. *The need to express love* refers to an important component of relationship maintenance, achieved by doing something for a significant person or by expressing love through gratitude, providing affection, or showing thankfulness.

Based on Erskine’s relational needs dimensions, Žvelc et al. (2020) developed an instrument for measuring relational needs satisfaction in both clinical and nonclinical populations. Authenticity, introduced as a new dimension, includes items related to Erskine’s needs for security, self-definition, and validation. The need for authenticity, or being authentic in a relationship, refers to a person’s ability to truly be themselves with another person while feeling respect, security, and understanding and that their individuality and personal uniqueness are accepted (Žvelc et al., 2020). However, Erskine’s need to express love was not included due to its nonrecognition as a separate dimension (Žvelc et al., 2020).

The final version of the Relational Needs Satisfaction Scale (RNSS) includes 20 items rated on a 5-point Likert scale from “agree” to “disagree” and represents five dimensions of relational needs: (1) Authenticity (e.g., “I can show my true self to people who are important to me without fear of rejection.”); (2) Support and Protection (e.g., “I have a strong, stable, and protective person in my life whom I can rely on.”); (3) Having an Impact (e.g., “Other people often ask for my opinion on certain topics.”); (4) Shared Experience (e.g., “I know people with a worldview similar to mine.”); (5) Initiative from the Other (e.g., “Other people sometimes surprise me in nice ways.”). Higher self-compassion, life satisfaction, emotional well-being, and attachment security are reflected in higher relational needs satisfaction (Pourová et al., 2020; Žvelc et al., 2020). The entire scale was highly reliable (*α* = 0.90), while the subscales had acceptable to good reliability: Authenticity (*α* = 0.80), Support and Protection (*α* = 0.85), Having an Impact (*α* = 0.81), Shared Experience (*α* = 0.73), and Initiative from the Other (*α* = 0.83).

The instrument has been validated in Slovenian (Žvelc et al., 2020), Czech (Pourová et al., 2020), Turkish (Toksoy et al., 2020), Spanish (Iraurgi et al., 2022), and Chinese (Yu et al., 2024), demonstrating adequate reliability and validity by relating to theoretically predicted constructs and replicating factor models. Moreover, a new instrument, the Workplace Relational Needs Satisfaction Scale (W-RNSS), has been developed and validated (Hanc et al., 2024), which enhances the understanding of how interpersonal factors impact crucial aspects of motivation in the work environment.

The perception that we are loved in a relationship stems from our own satisfaction of relational needs within that relationship (Žvelc et al., 2020). Responses to our relational needs are significant not only in childhood but also throughout all stages of life, resembling attachment styles in terms of the persistence of these needs (Erskine et al., 1999; Erskine, 2015). Considering the foundation of the relational needs model in attachment theory, recognizing which needs are unmet offers a clearer insight into relational challenges (Žvelc et al., 2020). Conversely, unmet relational needs may lead to cognitive defenses that manifest in self- and other-negative script beliefs (Erskine et al., 2023). To investigate whether, within our sample, attachment style and satisfaction of relational needs are linked, the Relationship Questionnaire (RQ) is used. The RQ (Bartholomew and Horowitz, 1991) is a tool that examines four types of attachment styles (i.e., secure, fearful, dismissive, and preoccupied) in the adult population.

Significant interpersonal relationships can trigger the expression of maladaptive personality traits. Furthermore, the specific individuals with whom we interact, those we contemplate, or members of our social networks can elicit varying expressions of normative personality dimensions (Lutz and Lakey, 2024). Hence, a strong need for drama contributes to crises across relationship types, leading to counterproductive behaviors and difficulties in social functioning (Ćubela Adorić et al., 2020). As the authors highlighted, these difficulties may stem from using maladaptive strategies to regulate unpleasant emotions in ourselves and others. Therefore, we hypothesized that maladaptive personality traits exploited in interpersonal relationships contribute to maladaptive relational patterns. To explore whether maladaptive traits are associated with relational needs satisfaction, we used the Need for Drama Questionnaire (NFD). The NFD questionnaire (Frankowski et al., 2016) measures the impulsive manipulation of others from a victimhood perspective, resulting from a combination of maladaptive personality traits (i.e., interpersonal manipulation, impulsive outspokenness, and persistent perceived victimhood).

Driven by the cross-cultural exploration of the RNSS instrument, as suggested by Žvelc et al. (2020), and the lack of instruments exploring Relational Needs Satisfaction in Bosnia and Herzegovina, the aim of the present study is to validate the RNSS version in Bosnian. We hypothesize that results similar to those from previously published validations and adaptations, which exhibit adequate psychometric properties, will be observed, providing new evidence regarding the instrument’s validity.

## 2 Materials and methods

### 2.1 Participants

The sample consisted of 420 participants from Bosnia and Herzegovina, of whom 71% were women (332) and 29% were men (87). The majority (62%) were between the ages of 27 and 44, although the sample’s age range was from 18 to 75. Regarding their education, 40.4% (171) completed their undergraduate degree, 28% (119) completed a graduate degree, 22.6% (95) had a high school diploma, 7.8% (33) held a PhD, and 0.5% (2) had a secondary school degree. Additionally, 80% (336) of them were married, 5% (22) were cohabiting, 7% (29) were in a relationship, 3% (12) were divorced, 4.5% (19) were single, and 0.5% (2) were widowed.

### 2.2 Instruments

#### 2.2.1 Relational needs satisfaction scale

The scale was developed by Žvelc et al. (2020) based on Erskine’s (2015) model of relational needs. It assesses the satisfaction of relational needs and consists of five subscales that represent the following relational needs: authenticity, support and protection, impact, shared experience, and initiative from the other. Scores can be calculated based on each subscale individually or as an overall score for relational needs satisfaction. The seminal study found the total scale is highly reliable (*α* = 0.90), as are the five subscales (α ranging from 0.73 to 0.85). In this study, we used the Bosnian translation of the scale, which includes 20 items on a 5-point Likert scale, where 1 signifies “never true,” and 5 denotes “always true,” with its reliability assessed at *ω* = 0.90.

#### 2.2.2 The relationships questionnaire

The Relationships Questionnaire (Bartholomew and Horowitz, 1991) is a four-item measure designed to assess the attachment style of adults. It presents participants with brief descriptions of four general relationship styles and asks them to choose the one that best describes their relating style. The participants were then asked to rate, on a 7-point Likert scale, how well each description corresponded to their relationship style. The attachment styles described are secure, fearful, dismissive, and preoccupied. Secure attachment: “*It is easy for me to become emotionally close to others. I am comfortable depending on them and having them depend on me. I do not worry about being alone or having others not accept me*.” Fearful attachment: *“I am uncomfortable getting close to others. I want emotionally close relationships, but I find it difficult to trust others completely or to depend on them. I worry that I will be hurt if I allow myself to become too close to others.”* Preoccupied attachment: *“I am uncomfortable getting close to others. I want emotionally close relationships, but I find it difficult to trust others completely or to depend on them. I worry that I will be hurt if I allow myself to become too close to others.”* Dismissive attachment: *“I am comfortable without close emotional relationships. It is very important to me to feel independent and self-sufficient, and I prefer not to depend on others or have others depend on me.”* The test–retest reliability of this measure ranges from 0.74 to 0.88 (Ligiéro and Gelso, 2002).

#### 2.2.3 Need for drama questionnaire

The Need for Drama Questionnaire (NFD), developed by Frankowski et al. (2016), measures a complex maladaptive trait characterized by the impulsive manipulation of others from a false victim position. The NFD consists of 12 items rated on a 7-point Likert scale, ranging from “strongly agree” to “strongly disagree.” It includes three dimensions: Interpersonal Manipulation (e.g., “Sometimes *I play people against each other to get what I want*”), Impulsive Outspokenness (e.g., “*It’s hard for me to hold my opinion back*”), and Persistent Perceived Victimhood (e.g., “*I feel like there are people in my life who are out to get me.*”). The total score on the scale, as well as its subscales, is calculated as the mean value of the designated items. In this study, the reliability of the total scale was *ω* = 0.85, while the reliability for the subscales ranged from 0.75 to 0.86.

#### 2.2.4 The sociodemographic questionnaire

The Sociodemographic Questionnaire was constructed for this study. It includes questions about sex, age, marital status, number of children, education, longevity of employment, salary, and history of mental health disorders.

### 2.3 Procedure

Upon approval for the adaptation of the Relational Needs Satisfaction Scale (RNSS, Žvelc et al., 2020), two independent translators translated the RNSS into a Bosnian and Herzegovinian version. Afterward, this version was provided to 10 (*n* = 10) integrative psychotherapists under supervision to receive qualitative feedback regarding word usage, ease of understanding of the statements, the extent to which the proposed statements represent what an individual may feel, and the relevance of the statements to the topic of relationships. Then, two other independent translators completed the back-translation. The translations were compared and unified. The author of the original scale checked the items and confirmed the translation. The second phase involved testing the psychometric properties, which included testing item properties, scale reliability, validity, and factor structure. Four hundred and twenty participants (*N* = 420) were recruited using a convenient snowball method from the general population of Bosnia and Herzegovina. An online version (Google Forms) of the instruments was distributed to them via email and social media. They were not compensated for their participation. Informed consent was obtained from all participants. The study is approved by the Institutional Review Board of the International University of Sarajevo (Ref. No. IUS-REC-01-01-3560/23).

### 2.4 Statistical analysis

For the RNSS scale, a descriptive statistical analysis was conducted, including means, standard deviations, frequencies, and percentages of responses for each option, as well as skewness, kurtosis, inter-item correlations, correlations of each item to the total score, and McDonald’s *ω* coefficient if the item was deleted. To explore the factor structure, we first checked the adequacy of the correlation matrix using the KMO test and Bartlett’s test of sphericity, followed by conducting exploratory factor analysis. To confirm the theoretical factor structure and identify the most appropriate model for the data, confirmatory factor analysis (CFA) was conducted. We tested one-factor, five-factor, hierarchical, and bi-factor models. The models were compared using several indices, including the normed chi-squared statistic (*χ*^2^/df), comparative fit index (CFI), Tucker-Lewis Index (TLI), root mean square error of approximation (RMSEA), standardized root mean square residual (SRMR), and Akaike information criterion (AIC). Measurement invariance was also examined for age based on the aforementioned indices. Convergent validity was assessed through correlation using the Pearson correlation coefficient between the RNSS scale and its subscales, as well as two reference instruments: the RQ and NFD scales and their subscales. The reliability of the measures was evaluated by assessing internal consistency using Cronbach’s alpha and McDonald’s omega coefficients. All analyses were performed using JASP software (version 0.19.0.0) and AMOS Graphics (version 20).

## 3 Results

An exploratory factor analysis was conducted after the conditions for its use were assessed to assess the factor structure. The value of Bartlett’s test of sphericity was significant (*χ*^2^ = 3,373, *p* < 0.001), and the Kaiser-Meyer-Olkin Measure of sampling adequacy was 0.89, indicating substantial correlation and the feasibility of conducting EFA. EFA, using the number of factors based on parallel analysis, provided a factor solution comprising six factors; however, the sixth factor consisted of a single item loading, which was higher on another factor. This solution explained 51% of the variance. EFA, based on eigenvalues, revealed a five-factor structure, with eigenvalues exceeding 1. The first factor had an eigenvalue of 6.56, the second factor had an eigenvalue of 1.8, the third had an eigenvalue of 1.33, the fourth had an eigenvalue of 1.16, and the fifth had an eigenvalue of 1.003.

To assess whether our data correspond with the theoretical structure and the structures proposed in previous research, we conducted a confirmatory factor analysis for the five-factor model, a single-factor model, a hierarchical model with five factors and one common factor (second-order CFA), and a bi-factor model where the items saturate both the five dimensions and a general factor simultaneously. The Maximum Likelihood method was used to estimate parameters, and the models were assessed using the following parameters: a normed chi-square statistic (*χ*^2^/df), a comparative fit index (CFI), a Tucker-Lewis Index (TLI), the root mean square error of approximation (RMSEA), the standardized root mean square residual (SRMR), and the Akaike information criterion (AIC). The following criteria for adequate model fit included a normed chi-square lower than or equal to three; an RMSEA value of 0.08 or below is considered a reasonably good fit, while a value below 0.05 indicates a close fit; an SRMR of 0.10 or lower is also considered a good fit; and CFI and TLI values between 0.90 and 0.95 are deemed acceptable. When comparing models, a lower AIC value suggests a poorer fit (Brown, 2015; Schermelleh-Engel et al., 2003, as cited in Žvelc et al., 2020). Table 1 shows the fit indices for all models, which are adequate for all except the one-factor model.

**Table 1:** Fit indices for confirmatory factor analysis.

Model	*χ* ^2^	df	*p*	CFI	TLI	SRMR	RMSEA	AIC
1-factor	1134.006	160	0.001	0.704	0.669	0.05	0.116	20558.435
5-factor	449	160	0.001	0.911	0.895	0.05	0.0656	19,893
Hierarchical	467.84	165	0.001	0.907	0.893	0.05	0.066	19902.207
bi-factor	434.067	150	0.001	0.913	0.889	0.05	0.067	19898.436

**Figure 1:**
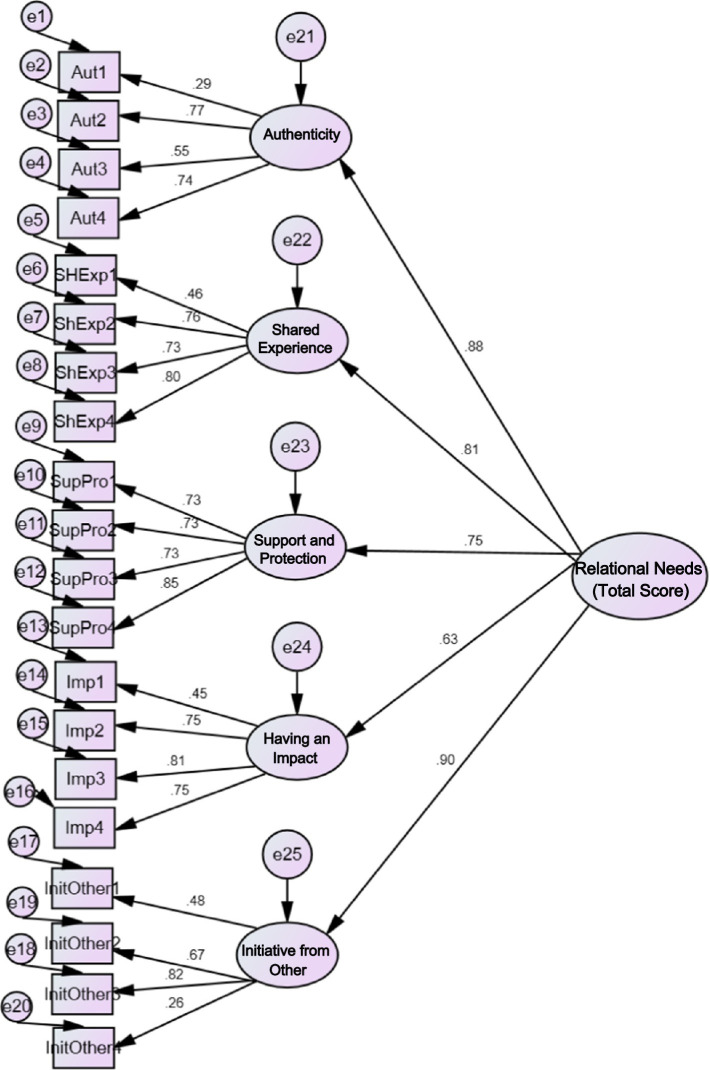
Illustration of the hierarchical model.

**Figure 2:**
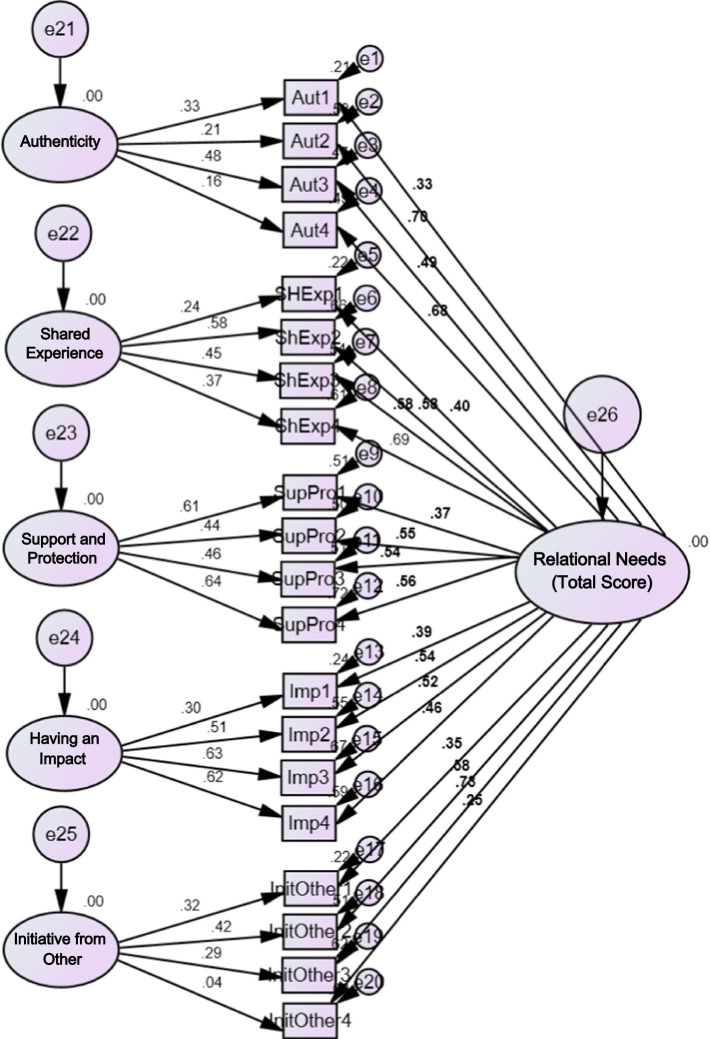
Illustration of the bi-factor model’s factor loadings.

Figure 1 shows the graphical representation of the hierarchical model, specifically the second-order confirmatory factor analysis factor loadings, which were conducted to determine whether the five factors could be subsumed under a second-order dimension. The figure includes the loadings of items on the five factors shown by the five-factor model, as well as the loadings of the five factors on a second-order factor of Relational Needs. Only Item 02 (Aut1, *λ* = 0.29) and Item 18 (InitiOther4, *λ* = 0.26) have low factor loadings (below 0.3). All second-order factor loadings are above 0.7, except for the saturation of the having an impact scale, which has *λ* = 0.63. Figure 2 shows the bi-factor model factor loadings, which tested the possibility of the RNSS item saturating both a general factor and its five subdimensions simultaneously. In general, the items tend to have either a higher loading on the general factor of Relational Needs or a very similar loading on both the general factor and the subscale. On the general factor, all items had loadings above 0.3, except for Item 18 (InitOther4, *λ* = 0.25). At the subscale level, only items 04 (Aut2, *λ* = 0.21), 12 (Aut4, *λ* = 0.16), 01 (ShExp1, *λ* = 0.24), and 18 (InitOther4, *λ* = 0.04) have loadings below 0.3. Based on the indices, it can be concluded that all models except the 1-factor model are viable; furthermore, the bifactor model aligns most closely with the theory.

We also evaluated whether relational needs were consistently measured across subsamples of our sample, particularly among different age groups, considering the wide age range in the sample. This was achieved by examining the invariance of the bifactor structure across two age groups within a structural equation modeling framework using confirmatory factor analysis. Widaman and Reise (1997, as cited in Putnick and Bornstein, 2016) propose four steps for testing measurement invariance. Configural invariance, which is the least stringent, assesses model form equivalence across groups, meaning that the same loadings are significant for all groups. Metric (weak factorial) invariance tests whether factor loadings are similar in size across groups. Scalar (strong factorial) invariance examines item intercept equivalence in all groups, while strict (residual) invariance, the most stringent, tests whether item residual variances are the same across groups. To assess the differences between these models, we analyzed the following indices: chi-squared differences (*χ*^2^), comparative fit index (CFI), and root mean square error of approximation (RMSEA). Configural invariance is evaluated by assessing the overall model fit, while the other models are tested by comparing each one to the previous model and analyzing the magnitude of the difference in fit statistics. *χ*^2^ is often considered too sensitive to small deviations in large samples, but a significant *χ*^2^ difference would indicate noninvariance. The CFI change criterion is commonly set at 0.01, while it is 0.015 for RMSEA (Chen, 2007); the difference values must exceed these benchmarks to conclude that the models are different and that non-invariance exists. Based on this criterion, the configural, metric, scalar, and strict models show no significant differences, leading us to conclude that invariance was established at all levels (Table 2).

**Table 2:** Fit indices for the invariance tests of the relational need satisfaction scale by age *N* (18–34) = 194, *N* (35–75) = 226.

Invariance	*χ* ^2^	df	Δ*χ*^2^	CFI	ΔCFI	RMSEA	ΔRMSEA
Configural	893.7	300		0.909		0.049	
Metric	866.1	334	2.760.000	0.918	0.009	0.044	0.005
Scalar	866.1	349	0	0.92	0.002	0.042	0.002
Strict	866.1	369	11.9	0.923	0.003	0.04	0.001

Tables 3, 4 show the descriptive data for all items: their means, standard deviations, skewness, percentage of choices per numerical option, correlations to both scale and subscale totals, McDonald’s *ω* values if the item was removed at the scale and subscale level, communality values, and factor loadings. The mean of the total scale is 3.95 on a range of 1 to 5. All items, except item 7, show negative skewness, indicating that participants tended to choose higher values, which is also reflected in the means. Two items in the Authenticity scale (12, 16) showed a possible ceiling effect, as did all the items in the Support and Protection scale (03, 04, 13, 17), with more than 50% of the respondents choosing option 5. No floor effects were observed. The reliability of the scale was tested using McDonald’s ω, given that the bifactor model was not essentially tau-equivalent. Under these conditions, using Cronbach’s alpha would underestimate reliability, and it is recommended to report McDonald’s ω (Padilla and Divers, 2015). The internal consistency of the entire RNSS scale was high (*ω* = 0.9), and the removal of any items would not result in a significant increase. The Support and Protection (*ω* = 0.85), Having an Impact (*ω* = 0.79), and Shared Experiences (*ω* = 0.79) scales also had high omega values. In contrast, the Authenticity (*ω* = 0.7) and Initiative from Other (*ω* = 0.66) scales had lower omega values. Although the removal of item 02 in the Authenticity scale would increase the reliability to 0.73, and the removal of item 18 from the Initiative from Other scale would increase its reliability to 0.7. The communality is low (less than 0.2) only for item 18 (*h*^2^ = 0.01), indicating that a very low proportion of variance in this item was explained by the five factors. Item 18 is also the only item that has an insufficient factor loading (less than 0.3), *λ* = 0.15 for its factor Initiative from Other. The skewness values for all subscales are well within the +2 to −2 range, indicating a normal distribution.

**Table 3:** Descriptive statistics and psychometric properties of RNSS.

	Percentage distribution					Descriptive statistics			
Item no.	1	2	3	4	5	M	SD	Sk	K
Authenticity						3.96	0.73	−0.75	0.44
02	6	13.6	30.5	36.2	13.8	3.38	1.07	−0.42	−0.36
11	1.9	6.4	18.8	36.2	36.7	3.99	0.99	−0.85	0.23
12	6	5.2	6.9	29.3	52.6	4.17	1.15	−1.51	1.45
16	1	3.3	11.4	33.8	50.5	4.30	0.87	−1.27	1.46
Support and protection						4.43	0.75	−1.55	2.2
03	2.4	6	8.8	18.6	64.3	4.36	1.03	−1.64	1.86
04	1	5.5	7.6	20.2	65.7	4.44	0.92	−1.71	2.25
13	0.5	1.9	5.5	21.9	70.2	4.6	0.72	−2.07	4.65
17	1.2	4.8	11.7	25.5	56.9	4.35	0.94	−1.38	0.51
Having an impact						3.67	0.67	−0.18	0.11
06	2.4	6.7	41.9	35.8	14.3	3.52	0.9	−0.23	0.13
15	2.9	5.5	34	38.8	18.8	3.65	0.94	−0.48	0.23
19	1.2	4.8	32.6	49.3	12.1	3.66	0.8	−0.46	0.57
20	0.5	2.9	28.8	46	21.9	3.86	0.80	−0.29	−0.13
Shared experience						4.04	0.69	−0.68	0.08
01	2.4	5.5	29.8	44	18.3	3.7	0.91	−0.58	0.42
05	1	4.3	16.4	36.2	42.1	3.7	0.91	−0.58	2.25
08	1	5.5	18.1	38.6	36.9	4.14	0.9	−1.71	0.2
14	0.5	2.6	15	35.2	46.7	4.05	0.92	−0.81	0.51
Initiative from other						3.64	0.72	−0.39	0.37
07	7.6	22.6	36.7	23.3	9.8	3.05	1.07	0.005	−0.57
09	1.4	5.5	26	43.1	24	3.83	0.91	−0.57	0.21
10	1.9	6.9	18.1	36.2	36.9	3.99	1	−0.86	0.21
18	6.2	11.4	32.8	25.2	33.3	3.68	1.22	−0.58	−0.64
RNSS total score						3.95	0.54	−0.57	0.07

**Table 4:** Descriptive statistics and psychometric properties of the RNSS.

Item no.	Reliability				EFA	
	Subscales		Scales			
	*r*	*ω*	*r*	*ω*	*h* ^2^	*λ*
Authenticity		0.7				
02	0.295	0.73	0.26	0.9	0.208	0.384
11	0.544	0.59	0.62	0.89	0.555	0.482
12	05	0.66	0.5	0.89	0.508	0.635
16	0.504	0.6	0.62	0.89	0.535	0.402
Support and protection		0.85				
03	0.68	0.81	0.51	0.89	0.602	0.714
04	0.66	0.82	0.61	0.89	0.634	0.64
13	0.63	0.82	0.56	0.89	0.578	0.669
17	0.75	0.77	0.61	0.89	0.72	0.791
Having an impact		0.79				
06	0.41	0.81	0.35	0.9	0.302	0.4
15	0.64	0.72	0.53	0.89	0.581	0.685
19	0.66	0.7	0.5	0.89	0.675	0.787
20	0.62	0.72	0.45	0.89	0.574	0.728
Shared experience		0.79				
01	0.41	0.81	0.39	0.89	0.239	0.37
05	0.66	0.7	0.6	0.89	0.72	0.75
08	0.63	0.72	0.57	0.89	0.572	0.627
14	0.64	0.71	0.67	0.89	0.661	0.626
Initiative from other		0.66				
07	0.39	0.63	0.43	0.89	0.338	0.407
09	0.48	0.53	0.57	0.89	0.524	0.574
10	0.53	0.48	0.67	0.89	0.605	0.53
18	0.21	0.7	0.23	0.9	0.095	0.15
RNSS total score				0.89		

To assess convergent validity, the RNSS was correlated with the Relationship Questionnaire, which measures attachment styles, and the Need for Drama scale, which evaluates maladaptive, manipulative, victim-playing relational styles. Table 5 shows that secure attachment has a significant positive correlation with all RNSS scales and the total score, although all correlations are low (0.13–0.23). Fearful attachment exhibits low but significant negative correlations with the RNSS total and all scales (0.13–0.24), except for Shared Experience, which is insignificant. Dismissive attachment shows no significant correlations with any of the scales or the RNSS total. Finally, the preoccupied attachment style was significantly negatively correlated with all scales and the RNSS total, though the correlations were low (−0.2 - -0.24). Table 5 also shows intercorrelations among the RNSS scales, all of which are significant at the 0.001 level and moderate, ranging from 0.3 to 0.54. Additionally, Table 5 shows that interpersonal manipulation has low but significant negative correlations with Shared Experiences, Support and Protection, Initiative from Other, and the RNSS total score (−0.14 to −0.21). Impulsive outspokenness shows a significant negative correlation only with Initiative from Other (−0.18). Persistent perceived victimhood exhibited low to moderate negative correlations with Authenticity, Support and Protection, Having an Impact, Shared Experiences, Initiative from Other, and the RNSS total score (−0.3 – -0.15). Finally, the total Need for Drama scale score demonstrated significant moderate negative correlations with Authenticity, Support and Protection, Shared Experience, Initiative from Other, and the RNSS total score (−0.29 – −0.18).

**Table 5:** Correlations between RNSS and attachment styles measured by the relationship questionnaire.

	Authenticity	Support and protection	Having an impact	Shared experience	Initiative from other	RNSS total
Relationship needs satisfaction scale						
Authenticity	–					
Support and protection	0.49***	–				
Having an impact	0.43***	0.3***	–			
Shared experience	0.49***	0.52***	0.45***	–		
Initiative from other	0.51***	0.51***	0.39***	0.54***	–	
RNSS total	0.78***	0.76***	0.69***	0.79***	0.78***	–
Relationship questionnaire						
Secure	0.18***	0.15**	0.23***	0.13**	0.17***	0.23***
Fearful	−0.24***	−0.16**	−0.13**	−0.1	−0.21***	−0.23***
Dismissive	−0.02	−0.04	−0.04	−0.1	−0.1	−0.1
Preoccupied	−0.18***	−0.21***	−0.21***	−0.16**	−0.2***	−0.24***
Need for drama scale						
IM	−0.09	−0.21**	0.01	−0.14**	−0.18***	−0.17***
IO	0.02	−0.12*	−0.04	−0.07	−0.18***	−0.10*
PPV	−0.26***	−0.20***	−0.15**	−0.23***	−0.28***	−030***
NFD total	−0.18***	−0.23***	−0.10*	−0.21***	−0.29***	−0,27***

*p < 0.05; **p < 0.01; ***p < 0.001, RNSS total, Relational Need Satisfaction Scale total score v; IM, Interpersonal manipulation; IO, Impulsive outspokenness; PPV, Persistent perceived victimhood; NFD total, Need for drama scale total score.

## 4 Discussion

The main objective of this study was to adapt the RNSS to the Bosnian version so that it could be further used in research and practice. The adaptation of the RNSS Bosnian version demonstrated content validity, adequate measurement accuracy, and appropriate construct validity, as supported by confirmatory factor analysis.

The metric characteristics of almost all items are acceptable, except for Item 02 in the Authenticity scale (“*I hardly have to hide anything in the company of people close to me.”*) and Item 18 in the Initiative from Other scale (“*No-one ever prepares a nice surprise for me.”*). Both items showed correlations below 0.3 with their respective scale’s total score and a potentially significant increase in reliability if removed, along with correlations below 0.3 with the overall score of the full scale. Item 02 contains double negative wording, which could make it unclear and create an answering bias. This issue could be addressed by using clearer wording. Item 18 is the only reverse-scored item, with possibly unclear wording, as it creates a double negative in translation. This item also requires rephrasing, possibly rewording it into a positive statement. Similar issues with Items 02 and 18, especially the latter, were identified in other language adaptations of RNSS (Iraurgi et al., 2022; Toksoy et al., 2020). Participants tended to select higher values on all items, most notably on the Support and Protection scale items, specifically Items 04 and 13. This negative asymmetry tendency was identical to the findings of Iraurgi et al. (2022). This finding was not surprising in psychological measurement due to social desirability and acquiescence bias (Crosswell and Lockwood, 2020; Tracey, 2016). Internal consistency, despite the poor performance of Items 02 and 18, was high regarding the total scale (*α* = 0.89) and would not improve with the removal of any items. This finding aligns closely with other studies, such as Iraurgi et al. (2022) adaptation to Spanish (*α* = 0.087), Turkish (Toksoy et al., 2020), and the original study in Slovenian (Žvelc et al., 2020; *α* = 0.90). The internal consistency for the subscales was lower than the values obtained in the original Slovenian study (Žvelc et al., 2020), but it was quite similar to what was observed in the Czech, Spanish, and Turkish adaptation studies (Iraurgi et al., 2022; Pourová et al., 2020; Toksoy et al., 2020). The Authenticity and Initiative from Other scales showed alpha values below 0.7, which is the acceptable threshold; however, it is important to note that these scales contain the previously mentioned problematic items, and their removal would bring the values closer to acceptability.

Another important aim of this study was to confirm the factor structure of the Relational Needs Satisfaction Scale. We first conducted an exploratory factor analysis, as we deemed the sample size adequate, according to Mundfrom et al. (2005). The exploratory factor analysis (EFA), based on parallel analysis, revealed a six-factor structure, as mentioned earlier, but the sixth factor was only saturated with one item that had a higher loading elsewhere. Only five factors had eigenvalues above 1, and the model explained 51% of the variance. The items were grouped according to theoretical predictions, meaning the appropriate items saturated the appropriate factors. Item 18 once again proved to be problematic, being the only item with insufficient factor loading. This, combined with reliability and total correlation issues of the scale, points to the need for a revision of this item. This finding adds to those of previous studies, indicating that it is not an isolated incident or a specific sample characteristic but rather a problem with the item.

We then attempted to confirm the factor structure and find the best model to explain the data. The original study proposed testing a five-factor model, a one-factor model, and a hierarchical model. Iraurgi et al. (2022) tested the bi-factor model but indicated a need for replication, so we also tested that model.

Table 5 shows that the worst-fitting model by all criteria, and the only model below all parameters of acceptability, is the 1-factor model. This model assumes only one general relational factor. A 1-factor solution contrasts with the theory of relational needs, which implies that there are multiple related, yet clearly distinct, needs that represent different aspects of the primary need for relatedness (Erskine, 2015). All three other models show very similar indices, all within the range of acceptability. The 5-factor model assumes five distinct, albeit correlated, factors representing relational needs. The hierarchical model brings us a step closer to the theoretical predictions suggesting that there are several distinct aspects of relational need, which can also be viewed as one general factor. This model predicts an overall factor composed of five lower-order dimensions. Higher-order CFA (Hierarchical CFA) is meant to provide a parsimonious explanation for the correlations among lower-order factors. It asserts that higher-order factors have a direct effect on the lower-order factors, which explains their covariation (Brown, 2015). The correlation matrix of our five factors shows that they are moderately to highly related, and the higher-order CFA indicates that this model fits the data. The bifactor model confirms that it is adequate to consider the items of RNSS as belonging, respectively, to the five dimensions of Authenticity, Support, and Protection, Having an Impact, Shared Experiences, and Initiative from Others while also belonging simultaneously to a general common factor of Relational Needs (the total score). In a model like this, there is a general factor that accounts for all the covariance in observed variables. However, there are also multiple domain-specific factors that, in addition to the general factor, explain the unique variance of the indicators. What distinguishes this model from the higher-order CFA used for the hierarchical model is that the bifactor model shows the direct effect of higher-order dimensions on the indicators. In these models, the lower-order dimensions and the general factor are specified as uncorrelated; hence, their contribution to explaining the unique variance of the indicators (items) is independent. The bifactor model fits best for constructs or measures that are essentially unidimensional but contain important subdomains that need to be accounted for to obtain a good model fit (Brown, 2015). The bifactor model appears to be the most suitable explanation for the observed data in this study, given Erskine’s (2015) theoretical prediction, supported and expanded upon by Žvelc et al. (2020), which validated a questionnaire indicating five dimensions and a general total factor simultaneously. The Spanish adaptation also found the bifactor model most fitting with both data and theory (Iraurgi et al., 2022). Additionally, as Chen et al. (2006) pointed out, it allows us to compare the significance of the general factor and the domain-specific second-order factors concerning a particular indicator. For instance, a domain-specific factor that previously contributed significantly to the indicator variance could become irrelevant with the introduction of the general factor to the model. In this study, the general factor and the domain-specific ones contribute equally to most items, with some exceptions that require further research beyond the current aims. We consider the bifactor model to be the most adequate, even though the fit indices are very similar to those of the hierarchical model and do not distinguish them. This is due to its ability to evaluate the degree to which item variance is explained by specific factors and a general one simultaneously, unlike the second-order CFA, which assumes that the general factor explains item variance only through the specific factors. Additionally, the bifactor model is more aligned with the theoretical underpinnings of RNSS, which conceptualizes it as an indicator of global relational need satisfaction, capable of distinguishing between specific needs simultaneously. Finally, the bifactor model was initially identified as the most adequate solution by Iraurgi et al. (2022), but it was recently replicated and confirmed by Hanc et al. (2024). We did not recommend the exclusion of any items, given that in a bifactor model, the items that have a loading below 0.3 on a specific factor have a higher and adequate loading on the general factor. The exception is Item 18 (IniOther04), which has a loading under 0.3 on both; however, this item has a wording problem caused by translation, as found in other studies. We believe it can be resolved by reformulation in future scale versions. We did not recommend the exclusion of items to maintain compatibility with scales in other languages. No previous studies recommended exclusion, only revision of problematic items.

Unfortunately, the study did not include a measure of discriminant validity due to time limitations and restrictions on sample availability; however, this should be examined in future studies.

Assessing the measurement invariance of the bifactor model across age groups showed that invariance was achieved. This means that the model is the same and fits the data well for all age groups. More specifically, the construct of interest, relational needs satisfaction, is interpreted in the same manner, regardless of the participants’ ages. This is congruent with the research of Hanc et al. (2024) and Pourová et al. (2020).

To assess convergent validity, we examined the relationship between the RNSS and attachment styles using the Relationship Questionnaire, as well as its relationship with the need for drama through the NFD scale. The findings regarding RNSS and attachment styles, which are very similar to those of the original study (Žvelc et al., 2020), show negative correlations with both preoccupied and fearful-avoidant styles, along with a positive correlation of the secure style with the RNSS subscales. In contrast, the dismissive-avoidant style showed insignificant correlations. This is consistent with the attachment literature (e.g., Lopez, 2009). A preoccupied style indicates a strong need for closeness with others, responsiveness from others, and the use of hyperactivating strategies to seek proximity. It appears logical that relational needs would be perceived as unmet in these individuals, given their intense focus on relationships and their craving to be as close as possible. This is reflected in the results of the present study, which indicate a negative correlation between the preoccupied attachment style and the RNSS total score. In contrast, a dismissive-avoidant style includes a tendency to deactivate proximity-seeking needs and behaviors, deny vulnerability, defensively maintain and enhance self-esteem, and divert attention from attachment-related threats. These individuals appear to suppress or deactivate relational needs; hence, the lack of correlations with RNSS in the current study aligns with this. However, the fearful-avoidant attachment style is neither clearly counter-dependent like the dismissive style nor desperately intimacy-seeking, similar to the preoccupied style. It involves a chronic conflict between the need for closeness and the fear of it, resulting in avoidance. Given this theoretical framework, it appears likely that their relational needs are still perceived as somewhat unmet and that correlations with relational needs would be slightly less than those of a preoccupied style but still present, unlike those of a dismissive style. This is reflected in our results, showing a negative correlation between the fearful avoidant attachment style and the RNSS total, which is almost identical in magnitude to the correlation with the preoccupied style. Finally, the secure attachment style’s positive correlations are explained by its characteristic readiness for seeking and engaging in relationships, as well as having more satisfying relationships. All of this indicates that their relational needs are well met (Lopez, 2009).

These findings support the convergent validity of the scale by displaying the theoretically expected correlation patterns with different attachment styles. The negative correlations between the preoccupied and fearful-avoidant attachment styles, along with the positive correlation between the secure attachment style and RNSS, illustrate that the scale identifies individual differences in how these needs are met or unmet. For those with a preoccupied or fearful-avoidant attachment style, these needs are perceived as severely unmet, as recognized by the scale, resulting in lower scores. Conversely, individuals with a secure attachment style perceive relational needs as mostly being met due to having satisfactory social relationships, leading them to achieve higher scores on the RNSS in this study, as also indicated by the scale. Therefore, there is supporting evidence that the scale meets the criteria for convergent validity by assessing the degree of perceived satisfaction of relational needs.

Our results revealed that the need for drama as an interpersonal trait is associated with relational needs, while interpersonal manipulation, impulsive outspokenness, and persistent perception of victimhood are related to maladaptive relational patterns. Specifically, interpersonal manipulation showed a negative association with relational needs for support and protection, shared experiences, and initiative from others, indicating that an increase in interpersonal manipulation leads to a decrease in perceived support, protection, shared experiences, and initiative from others. This can be explained by evidence suggesting that interpersonal manipulation may be viewed through the lens of how an individual approaches relationships and influences others, a pattern characterized by negligence and a lack of care (Klenk, 2021). The extent of self-oriented concern may diminish the degree of care for the welfare of others in a relationship, contributing to lower relationship well-being (Le et al., 2018). Moreover, the relationship between emotional manipulation and emotional intelligence may be moderated by psychopathy and narcissism (Nagler et al., 2014). A negative correlation is also found between impulsive outspokenness and relational needs for support and protection, as well as initiative from others, indicating that heightened impulsive outspokenness leads to a decrease in perceived support, protection, and initiative from others in relationships. Emotional processing may contribute to impulsivity and impulsive outspokenness, as impulsive behaviors can be triggered by emotions or occur independently of them (Carver and Johnson, 2018). Degrees of impulse control may be understood through associations with emotional dependence and a preoccupied attachment style, with emotional dependence serving as a mediator in this relationship. Estévez et al. (2018) highlighted that an increase in impulsivity contributes to a heightened perception of asymmetry in relationships, the need to please others, childhood trauma, and parental permission. From this perspective, impulsivity may play a role in how individuals perceive asymmetry in a relationship, as those with higher impulsive outspokenness are more likely to feel that they receive less support, protection, and initiative from others.

Moreover, our study shows the strongest associations between persistent perceived victimhood and all five relational needs. Specifically, an increase in persistent perceived victimhood significantly contributes to a decrease in experienced authenticity, support, and protection, which affects shared experiences and initiative from others in a relationship. The perception of oneself as a victim across various interpersonal relationships reflects personality tendencies with meaningful and stable traits, such as moral elitism, rumination, lack of empathy, and a need for recognition (Gabay et al., 2020). Furthermore, it impacts intrapersonal processes of biased self-perception and memory (Young and Sullivan, 2016), whereas individuals who perceive significant others as particularly critical tend to persistently feel victimized (Demirsen Ayar, 2023).

The correlation between maladaptive personality traits and relational needs is not surprising. Individuals with manipulative and impulsive tendencies may perceive these traits as self-efficacy, which perpetuates cycles of drama in their lives (Lue and Frankowski, 2020). Unequal power in relationships often signals lower trust and increased conflict (Du Plessis et al., 2023). Additionally, individuals who perceive themselves as powerful are more likely to seek social connections (Narayanan et al., 2013). The concept of interpersonal victimhood is shaped by attachment styles and cultural context, with a focus on a lack of empathy, rumination, and a need for recognition (Manolov, 2023), whereas perceived victimhood may lead to emotional distancing (Jasini et al., 2017).

Overall, individuals with a high Need for Drama (NFD) tend to have lower levels of self-esteem (Lue and Frankowski, 2020), with self-esteem having a stronger predictive ability for satisfaction in relationships than assertiveness (Moss et al., 2021). Moreover, we believe that relational needs satisfaction in future endeavors may be observed in line with the Big Five personality traits, considering that individuals who score higher in NFD, which refers to maladaptive personality traits, appear to be more neurotic, disagreeable and lack conscientiousness. This may be a useful predictor of various maladaptive interpersonal interactions in different contexts (Frankowski et al., 2016). Persistent concerns regarding self-image goals contribute to the perception of others as interpersonally colder. Moreover, when lower relational perceptions are present, individuals tend to recognize self-image goals as more important (Huang et al., 2023).

In summary, the adaptation of the RNSS Bosnian version demonstrated content validity, adequate measurement accuracy, and appropriate construct validity, as supported by confirmatory factor analysis indices. This study provides evidence that the translated Bosnian version of the RNSS is an appropriate instrument that can be used to assess relational needs in research and clinical practice. However, items 02 and 18 need linguistic revision, after which their characteristics need to be reexamined. Several limitations of the study should be noted: the sample size and type (general population, too homogeneous by gender and age) may limit the generalizability of the findings. The study relied on self-report data, which are subject to social desirability bias, especially in psychological research. Further research may explore predictive validity in association with functionality in clinical samples, as well as the comparative performance of the instrument across different samples. Discriminant validity needs to be examined, and additional ways of testing convergent validity ought to be applied. It may be useful to assess the RNSS relationship with more variables indicating relationship satisfaction and outcomes. Moreover, the utility of the RNSS as a diagnostic and guidance tool in psychotherapy across different approaches should be explored. The language adaptation process has indicated that the psychometric properties are met, concluding that the Bosnian adaptation of the RNSS has adequate characteristics for adaptation and measurement in the Bosnian and Herzegovinian languages. The availability of this instrument in Bosnian may facilitate research in the mentioned realms in Bosnia and Herzegovina. Future studies should further aim to replicate these findings in diverse cultural contexts to enhance the instrument’s global applicability.

## Data Availability

The original contributions presented in the study are included in the article/supplementary material, further inquiries can be directed to the corresponding authors.
